# Evidence of Local Structural Variations and Their Influence on Magnetic Properties in Mn- and Cr-Containing High-Entropy Oxide Thin Films Using Electron Microscopy

**DOI:** 10.1021/jacs.6c00090

**Published:** 2026-05-28

**Authors:** Sai Venkata Gayathri Ayyagari, Matthew Webb, Jacob T. Sivak, Gerald Bejger, John P. Barber, Debangshu Mukherjee, Kevin M Roccapriore, Aleksander B. Mosberg, Leixin Miao, Jon-Paul Maria, Susan B. Sinnott, Christina M. Rost, Quentin M. Ramasse, John T. Heron, Nasim Alem

**Affiliations:** 1 Department of Materials Science and Engineering, 8082 The Pennsylvania State University, University Park, Pennsylvania 16802, United States; 2 Department of Materials Science and Engineering, 1259 University of Michigan, Ann Arbor, Michigan 48109, United States; 3 Department of Chemistry, 8082 The Pennsylvania State University, University Park, Pennsylvania 16802, United States; 4 Department of Materials Science and Engineering, Virginia Polytechnic Institute and State University, Blacksburg, Virginia 24061, United States; 5 Computational Sciences & Engineering Division, 6146 Oak Ridge National Laboratory, Oak Ridge, Tennessee 37830, United States; 6 Center for Nanophase Materials Sciences, 6146 Oak Ridge National Laboratory, Oak Ridge, Tennessee 37830, United States; 7 AtomQ, Knoxville, Tennessee 37931, United States; 8 SuperSTEM Laboratory, SciTech Daresbury Campus, Daresbury WA4 4AD, U.K.; 9 Institute for Computational and Data Sciences, 8082 The Pennsylvania State University, University Park, Pennsylvania 16802, United States; 10 School of Chemical and Process Engineering & School of Physics and Astronomy, University of Leeds, Leeds LS2 9JT, U.K.

## Abstract

Alloying is an age-old strategy for synthesizing materials with enhanced properties. Recently, multicomponent systems such as high-entropy oxides have garnered widespread attention due to their tunable and often superior properties compared to their constituent oxides. Here, we study the local structural and chemical nuances of six-component (Mg_0.167_Co_0.167_Ni_0.167_Cu_0.167_Zn_0.167_Mn_0.167_)O and (Mg_0.167_Co_0.167_Ni_0.167_Cu_0.167_Zn_0.167_Cr_0.167_)O thin films. The Mn-alloyed thin film exhibits a higher exchange bias and greater magnetic frustration compared with the Cr-containing thin film. Scanning/transmission electron microscopy investigations reveal that the Mn-alloyed thin film exhibits the coexistence of rock salt and spinel-like regions, unlike the single-phase rock salt structure observed in the Cr-alloyed thin film. Electron energy loss spectroscopy indicates changes in Co and Mn valences within the Mn-containing thin film, suggesting the presence of mixed-valence states, which are further confirmed by X-ray absorption spectroscopy measurements. These observations are further validated by cation-site-preference energy calculations using density functional theory. Our results demonstrate how the chemistry, site occupations, and cation valences result in pronounced changes in the overall properties of high-entropy oxides.

## Graphical abstract

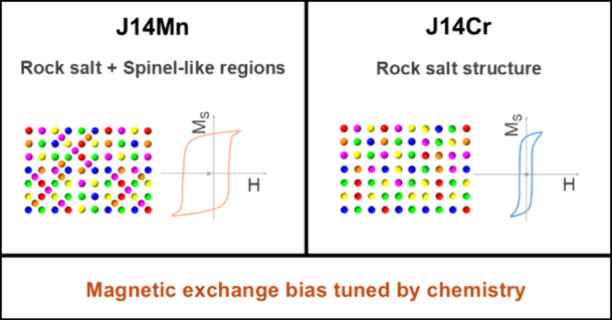

## Introduction

High-entropy oxides (HEOs) are a class of crystalline systems composed of five or more components occupying the cation sublattice site, forming an oxide solid solution. The first synthesized HEO is the prototypical five-component rock salt oxide, (Mg_0.2_Co_0.2_Ni_0.2_Cu_0.2_Zn_0.2_)O (referred to as J14 for brevity).^1^ Since then, various compositions and crystal structures of HEOs have been synthesized.^2^ It has been demonstrated that pulsed laser deposition (PLD), by accessing a regime of high effective quenching rates, enables the synthesis of compositions with misfit cations such as J14+Ca, J14+Sc, which could not be stabilized using conventional solid-state synthesis techniques, even at high temperatures.^3^ In recent years, reports have also shown that the microstructure and cation valences are strongly influenced by growth kinetics,^4–8^ including oxygen partial pressure, substrate temperature, laser repetition rate, and growth duration, thereby enabling tunable properties.^6^ These multicomponent systems with structural and chemical disorder have shown promise in a wide range of applications, including ionic conductors,^9^ transparent conductors,^10^ electrocaloric materials,^11,12^ thermoelectric materials,^13,14^ catalysts,^15^ and more.^16^

There is a growing interest in exploring the magnetic properties of HEOs due to spin disorder resulting from the mixing of magnetic and nonmagnetic cations, as well as complex magnetic correlations arising from chemical disorder.^17,18^ It has been reported that J14 exhibits a long-range antiferromagnetic order below the Néel temperature of 113 K, despite the presence of 40% nonmagnetic ions in the cation sublattice.^17,19,20^ Studies have shown that if the magnetic elements are not overly diluted and magnetic frustration is not too strong, the long-range magnetic order can be preserved.^21^

In this study, we investigate the structural nuances, chemical environment, and magnetic properties of six-component HEO thin films grown using PLD. We employ scanning transmission electron microscopy (STEM) and virtual dark-field imaging reconstructed from 4D STEM data, along with electron energy loss spectroscopy (EELS) and X-ray absorption spectroscopy, to understand the local structural and electronic variations. Exchange bias studies are carried out to characterize the exchange interaction of these antiferromagnetic HEO thin films with a soft ferromagnet.^22,23^ The magnitudes of the bias and coercive fields are influenced by magnetic frustration in the antiferromagnetic layer, allowing us to probe the magnetic properties of the antiferromagnetic HEOs.

Here, we observe that the thin film alloyed with Mn exhibits a higher exchange bias compared to that of Cr-alloyed J14. Local structural investigations reveal that the addition of Mn to J14 leads to the coexistence of rock salt and spinel-like regions, whereas the addition of Cr to J14 results in a purely rock salt structure. A significant change in the Co and Mn valences within the thin film is observed, which is attributed to these local structural changes.^24^ This observation is further supported by first-principles calculations of cation-site-preference energies, indicating that Mn tends to occupy both tetrahedral and octahedral sites depending on surrounding cations, leading to the formation of spinel-like regions. These findings suggest that the free energy landscape of the HEOs possesses multiple local minima, which can be accessed by nonequilibrium growth conditions by trapping different local structures and cation valences, offering opportunities to tune material properties.

## Results

### Thin Film Growth, Structure, and Magnetic Characterization

Two high-entropy oxide (HEO) thin films with compositions (Mg_0.167_Co_0.167_Ni_0.167_Cu_0.167_Zn_0.167_ **Mn**_0.167_)O and (Mg_0.167_Co_0.167_Ni_0.167_Cu_0.167_Zn_0.167_ **Cr**_0.167_)­O, hereafter referred to as J14Mn and J14Cr, respectively, are investigated to correlate their structure with their magnetic properties. These thin films are synthesized using PLD along the [001] direction at a constant temperature on [100] MgO substrates, with a thickness of approximately 60–65 nm. Pendellösung fringes, indicating the crystallinity of these thin films, are observed in the XRD pattern centered on the 200 peak of the MgO substrate, as shown in Figure S1a,b. From the XRD analysis, the out-of-plane lattice parameters of J14Mn and J14Cr are 4.15 and 4.17 Å, respectively. For magnetic studies, an exchange bias-based approach is utilized, requiring a layer of permalloy to be deposited on top of the HEO film and capped with a thin Pt layer, as shown in Figure 1a, since the high-entropy sample is expected to be antiferromagnetic. Cross-sectional STEM images in Figure 1b,c confirm the formation of epitaxial thin films. Fourier transform analysis of the STEM images reveals additional reflections in J14Mn that are absent in J14Cr, indicating local structural differences. These variations are further analyzed in this study.

**Figure 1:**
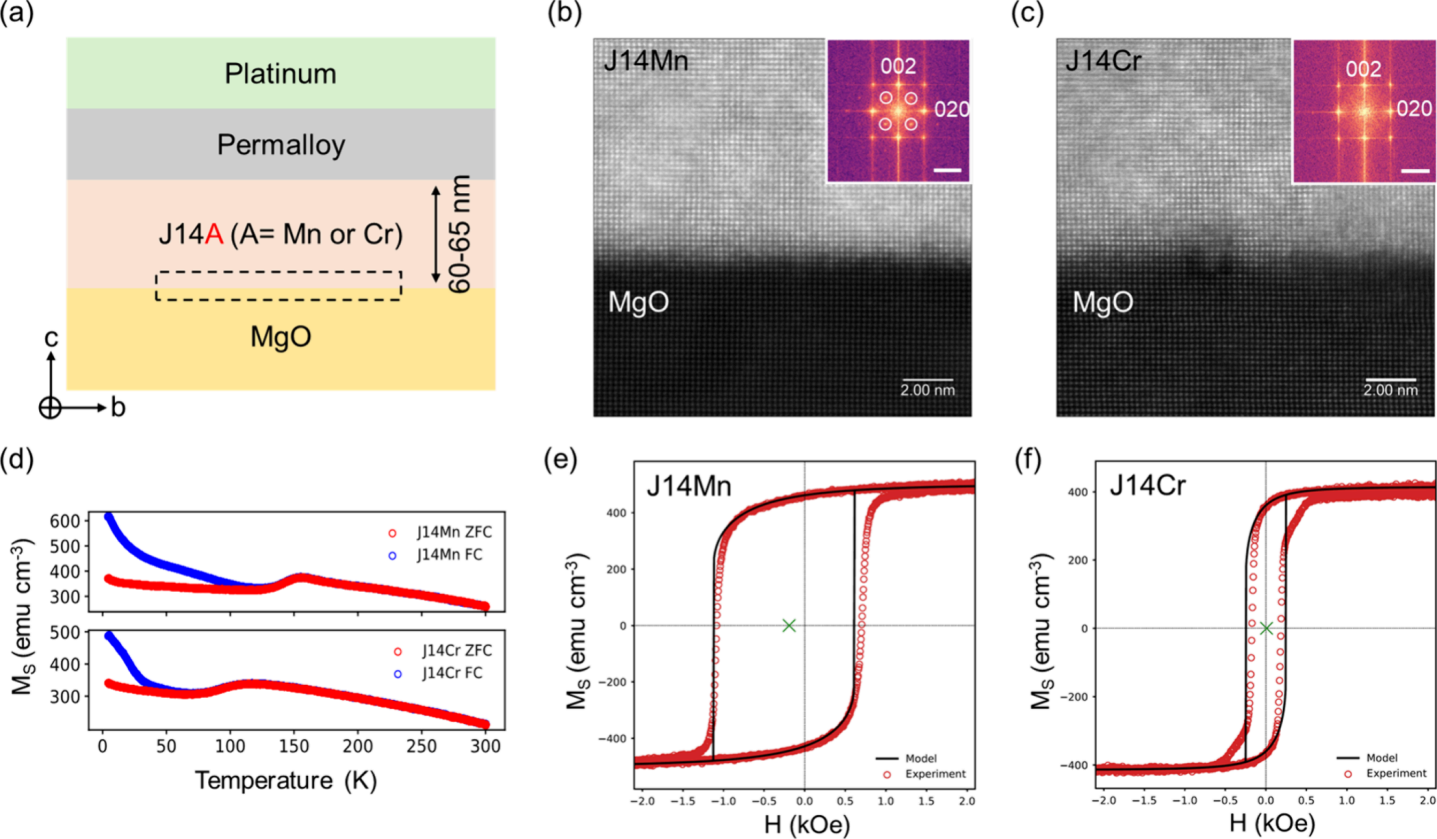
Overview of structure and magnetic properties: (a) schematic of the stacking sequence of the samples investigated in this study, where Pt and permalloy layers are deposited for magnetic studies. The dashed inset highlights the interface imaged in panels (b) and (c). (b,c) Atomic-resolution STEM images of J14Mn and J14Cr acquired along [100] zone axis, with their corresponding fast Fourier transforms (FFTs) shown in the insets. Additional reflections in the FFT of J14Mn are marked with circles. The scale bar in both insets in panels (c) and (d) is 5 nm^–1^. (d) Temperature-dependent magnetization (M-T) curves of J14Mn and J14Cr measured under field-cooled (FC) and zero-field-cooled (ZFC) conditions. (e,f) Exchange-biased magnetic hysteresis loops measured at 25 K along [100] crystallographic direction after cooling to in a 1 T field of J14Mn and J14Cr, respectively.

Magnetic measurements conducted on J14Mn and J14Cr indicate antiferromagnetic behavior. The temperature-dependent magnetization studies of these samples, measured under zero-field cooling (ZFC, 50 Oe) and field cooling (FC) with a magnetic field of 2000 Oe, are shown in Figure 1d. The curve shape clearly indicates that both thin films exhibit antiferromagnetic properties, with Néel temperatures of ∼120 K for J14Cr and ∼155 K for J14Mn. These Néel temperatures fall within the range reported in the literature for rock salt HEOs.^25^ The larger exchange bias exhibited by J14Mn, as observed in the wider and more shifted hysteresis loop, indicates that there is a greater antiferromagnetic ordering at this temperature compared to the J14Cr sample, which exhibits almost no exchange bias, indicating a highly disordered magnet.^22,23^ Further investigation shows that the blocking temperature, defined as the temperature at which the antiferromagnetic material loses the ability to pin a ferromagnetic layer, is 17 K for J14Cr and 60 K for J14Mn.

### Local Structural Investigation of J14Mn HEO

To examine the influence of Mn addition to J14 on the structure, we first investigate both reciprocal and real space using selected area electron diffraction (SAED) and HAADF-STEM imaging, respectively. The SAED pattern from the [100] zone axis, obtained from the J14Mn thin film and the MgO substrate, is presented in Figure 2a. The selected area contributing to this electron diffraction is shown in the inset in Figure 2a. The SAED pattern of J14Mn reveals additional reflections at *g̅* = {011} (marked with arrows in Figure 2a), which are typically forbidden in rock salt crystal structures.

**Figure 2:**
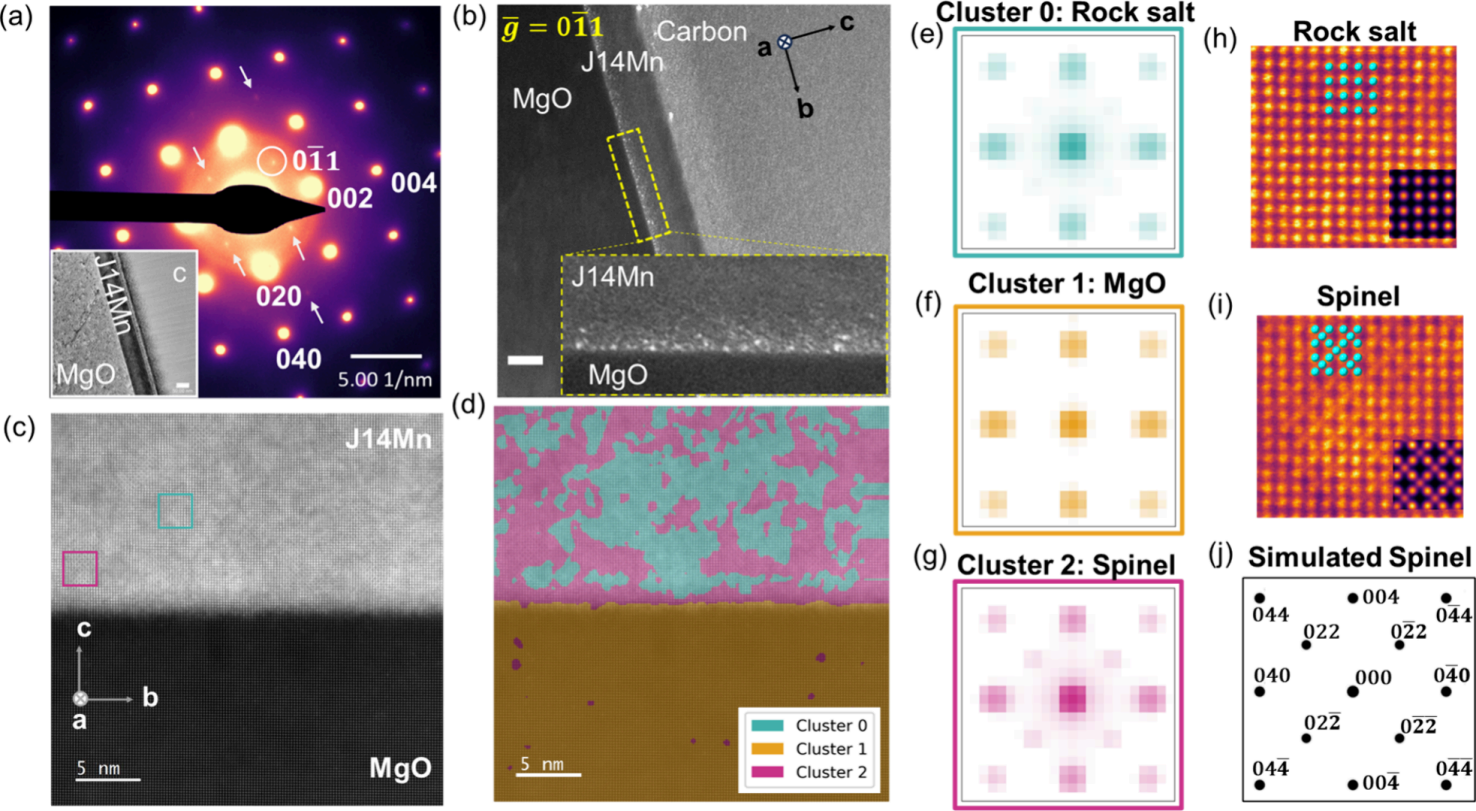
Local structure investigation of J14Mn HEO: (a) selected area electron diffraction (SAED) pattern of J14Mn acquired along the [100] zone axis, along with the corresponding selected area region (scale bar: 50 nm). Arrows indicate additional reflections typically absent in the rock salt structure. The circular inset corresponds to the dark-field TEM image shown in panel (b). Scale bar in panel (b) is 50 nm. The additional reflections predominantly originate from the interface between the thin film and MgO. (c) Atomic-resolution HAADF-STEM image of the interface. (d) Result of the image processing on panel (c) by implementing an unsupervised ML approach on the sliding window FFT. The local FFT patterns were categorized into three classes, corresponding to three distinct average FFTs. (e–g) Average FFT corresponding to each class in panel (d). (h,i) Higher-magnification STEM images of rock salt and spinel regions in J14Mn, with a magma color overlay, together with the corresponding simulated STEM images of these structures. (j) Simulated electron diffraction of spinel structure.

To understand the origin of these extra reflections, dark-field TEM (DF-TEM) imaging was performed using the extra reflection at *g̅* = 0 1̅ 1. The DF-TEM results indicate that the secondary phase is predominantly located at the interface between the J14Mn thin film and the MgO substrate, as shown in Figure 2b. To further probe these reflections, virtual dark-field (VDF) imaging was carried out on the 4D STEM data across the thin film, as shown in Figure S2, confirming that the additional reflections originate mainly from the interface between the thin film and the substrate. Finally, to investigate the atomic arrangement responsible for the extra reflections, atomic-resolution HAADF-STEM imaging was performed at the interface.

The HAADF-STEM image reveals local variations in the atomic arrangement, as highlighted by boxes in Figure 2c. To classify the local structural variation in Figure 2c, local symmetry analysis using unsupervised machine learning on the 2D FFT (see the Experimental Section/Methods section) was implemented. The image is categorized into three classes, with each color in Figure 2d representing a distinct class. The class-averaged FFT patterns corresponding to each class are presented in Figure 2e–g. The FFT with extra reflections (Figure 2g) matches closely with the simulated spinel electron diffraction (Figure 2j), indicating the presence of nanoscale spinel phases along with the rock salt structure. From the FFT and SAED analysis, we determine that the orientation relationship between the two phases is 044_spinel_ parallel to 022_rock salt_, with the lattice parameter of the spinel phase being twice that of the rock salt phase. We note that fainter extra reflections can be observed in the cluster 0 FFT (Figure 2e), which we attribute to slight overlap of spinel-like regions during the sliding window and clustering process; this effect was unavoidable.

Magnified HAADF-STEM images acquired from the vicinity of the interface, highlighting the different atomic periodicities in the thin film, are shown in Figure 2h,i. Figure 2h exhibits a rock salt-type arrangement, whereas Figure 2i reveals periodic columns of tetrahedrally coordinated cations, confirming a spinel-type crystal structure typical of Co_3_O_4_. The insets in Figure 2h,i correspond to simulated STEM images of rock salt MgO and spinel Co_3_O_4_ respectively, which closely reproduce the atomic arrangements observed in the material. The coexistence of rock salt and spinel phases has been previously reported in various compositions.^8,26–28^ Similar features were also observed in the prototype HEO J14 when the thin film was grown with increased thickness and at a faster growth rate.^8^

### Local Oxidation-State Investigation of Cations in J14Mn HEO

To investigate the oxidation state of cations at the nanoscale regime and how the presence of multiple cations or the formation of secondary phase alter the valences, EELS was performed. Our EELS measurements show a significant change in the edges of Mn, Co, and O across the J14Mn film, while Ni and Cu do not show any change in their fine structure (see SI, Figure S3). In EELS, electron interactions with core and inner-shell electrons contribute to the core-loss region (>50 eV) of the spectrum. The energy loss near edge structure (ELNES) in this core-loss region depends on coordination, valence state, and bonding. Any change in the ELNES shape or edge onset indicates a modification in the local atomic environment. The ELNES features for the Mn and Co L edges show a shift in energy loss and a shape change that are consistent with a deviation from the pure 2+ valence state to a mixture of 2+ and 3+ valence states for Mn and Co ions as we approach the interface.^29–31^ To correlate the shift in the Mn and Co L-edge with the local structural variations observed in the J14Mn HEO, we performed atomic-resolution EELS to further investigate whether the valence change is associated with the rock salt rather than spinel structures.

The summed ELNES of the Co L-edge from the rock salt and spinel regions, along with reference spectra of Co from CoO and Co_3_O_4_,^31^ are plotted in Figure 3a. A noticeable change in peak shape and position is observed. The integrated intensity ratio of the L_3_ and L_2_ peaks is related to the unoccupied states in the 3d level, making it a useful parameter for determining the valence state.^32^ From the Co L_3_/L_2_ intensity ratio (SI, Figure S5), the “average” Co oxidation state is ∼2.1+ in the rock salt phase (Co L_3_/L_2_ ratio ∼4.4), whereas it is approximately 2.66+ in the spinel region (Co L_3_/L_2_ ratio ∼3.3).^32^ Similarly, the summed ELNES of Mn L-edge, along with reference spectra from MnO and Mn_2_O_3_,^30^ are plotted in Figure 3b. The Mn L_3_/L_2_ ratios in the rock salt and spinel regions are ∼2.8 and 2.5 and inferring net valences of ∼ 2.5+ and 2.7+, respectively.^32^ This suggests that Co and Mn ions exhibit a mixed 2+ and 3+ valence state in the nanoscale spinel regions, resembling Co_3_O_4_ and Mn_3_O_4_ respectively. Besides the Co and Mn edges, we also observe evidence of the oxidation-state change in the O K-edge at around 529 eV in Figure 3c. Since the O K-edge reflects the hybridization of O 2p orbitals with transition-metal 3d states, any variation in the O K-edge indicates changes in the 3d orbitals of the transition-metal cations,^33,30^ which is consistent with the observed alterations in Co and Mn 3d states observed here.

**Figure 3:**
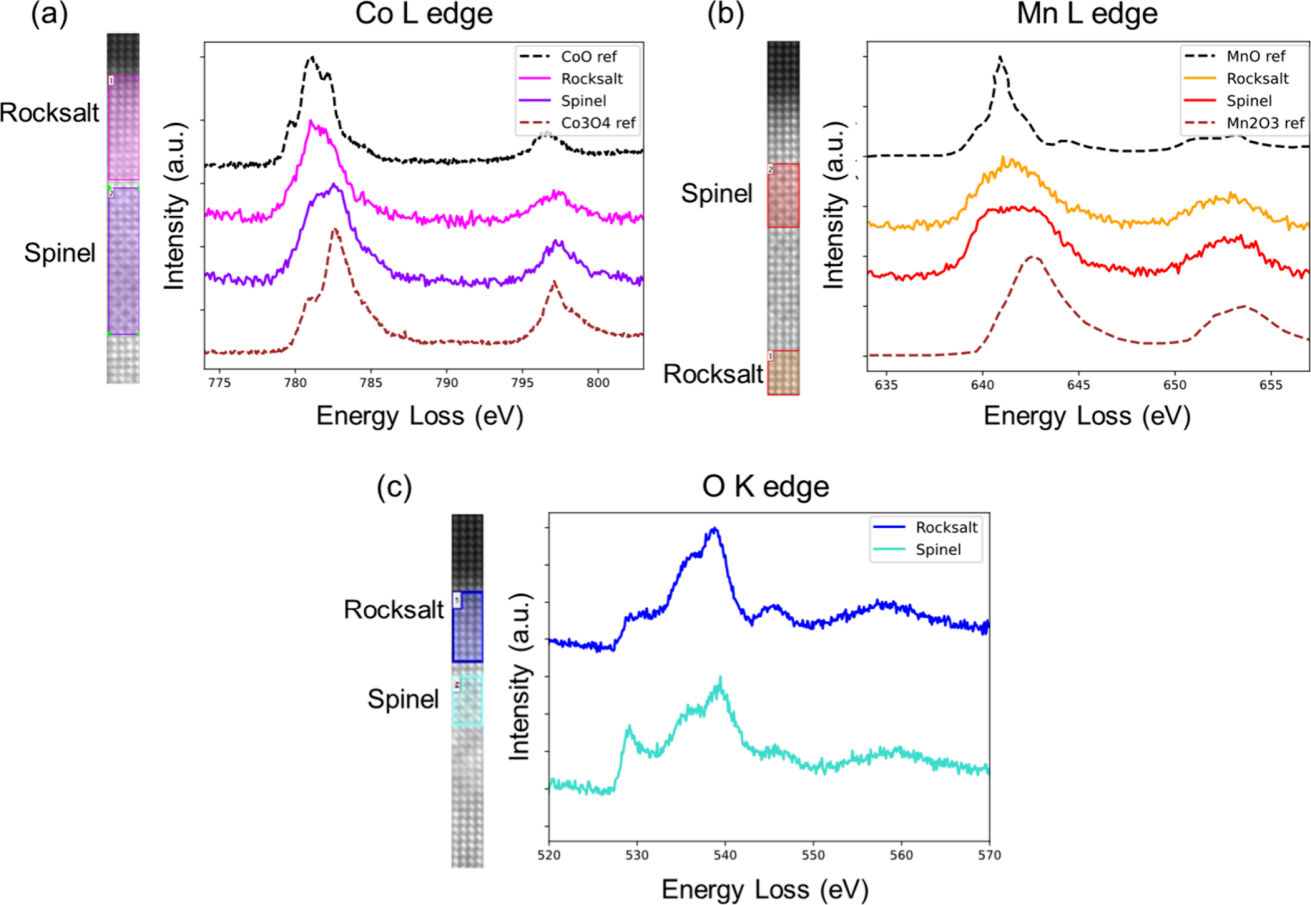
Investigation of oxidation states in J14Mn HEO: (a) Co L-edge along with CoO and Co_3_O_4_ references.^31^ (b) Mn L-edge along with MnO and Mn_2_O_3_ references.^30^ (c) O K-edge showing variation in oxidation states in spinel vs rock salt regions, and this change can also be seen as change in bonding environment for O.

### Local Structural Investigation of J14Cr HEO

Electron diffraction and HAADF-STEM imaging and analysis were carried out to understand the structure of J14Cr HEO. The SAED pattern from the [100] zone axis, obtained from the J14Cr thin film and MgO substrate, is presented in Figure 4a. The selected area contributing to this electron diffraction is shown in Figure 4b. The SAED pattern confirms the formation of a rock salt structure for both MgO and J14Cr thin films with no additional reflections, indicating the absence of any secondary phases or precipitates in the thin film. Additionally, the SAED pattern shows no discernible lattice mismatch between the thin film and the substrate.

**Figure 4:**
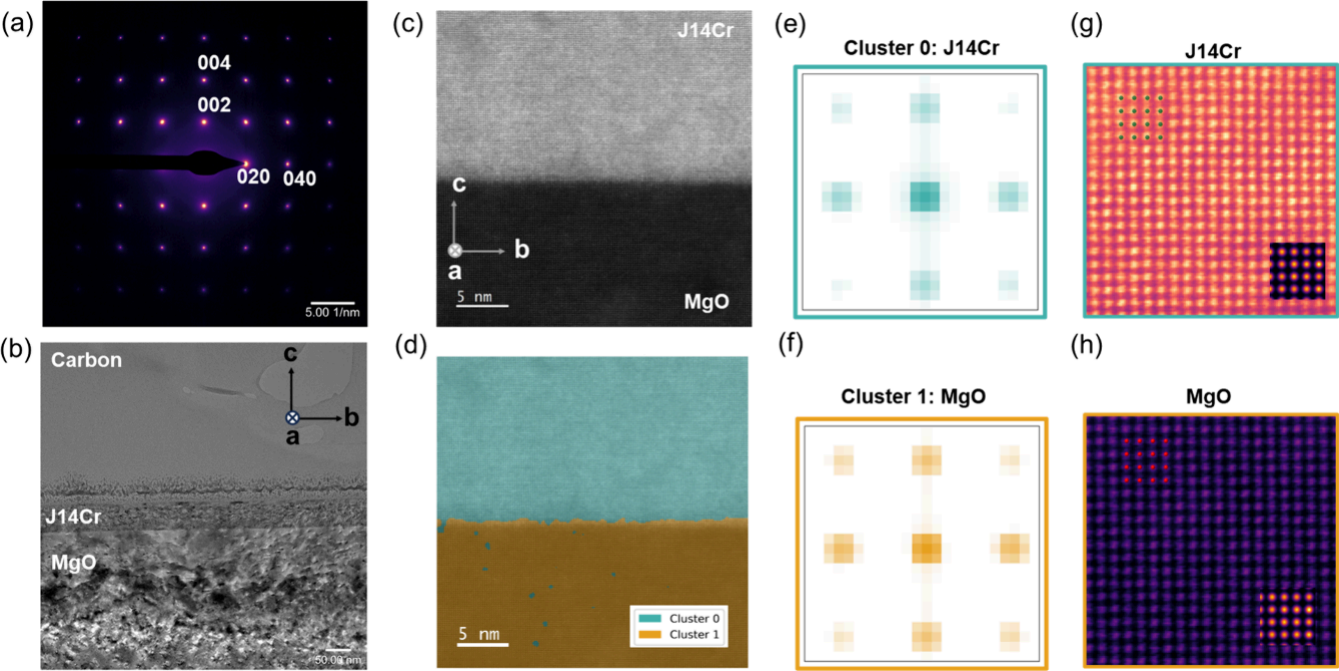
Local structure investigation of J14Cr HEO: (a) SAED pattern of J14Cr along [100] zone axis, showing the presence of a rock salt structure with no additional reflections. (b) Corresponding selected area image. (c) Atomic-resolution HAADF-STEM image of the interface. (d) Result of image processing on (c) using unsupervised machine learning applied to the sliding window FFT, which categorized the local FFT patterns into two clusters based on local crystallographic information. (e,f) FFT patterns corresponding to each cluster in panel (d). (g,h) Higher-magnification STEM image of J14Cr and MgO, with a magma color overlay and simulated STEM image of the structure, respectively. The magma color overlay was obtained from a single acquisition and cropped to show each region; therefore, the intensity scale is identical for both images, and the darker contrast in MgO reflects its lower average atomic number relative to J14Cr.

To further probe the structure, HAADF-STEM imaging was performed, with high-magnification STEM images shown in Figure 4c,g,h. Local symmetry analysis (Figure 4d–f) categorizing the data into two distinct classes confirms the formation of a rock salt structure with no additional structural variations.

### Local Oxidation-State Investigation of Cations in J14Cr HEO

Core-loss EELS experiments across the J14Cr thin film show no evident change in the peak positions of any cations in the J14Cr HEO thin film. The ELNES of the Co L-edge in J14Cr matches well with the CoO ELNES,^31^ as shown in Figure 5a. Hence, the Co oxidation state in J14Cr was determined to be 2+. Similarly, our analysis shows that the Cr L-edge aligns well with the Cr_2_O_3_ reference spectrum,^34^ as shown in Figure 5b.

**Figure 5:**
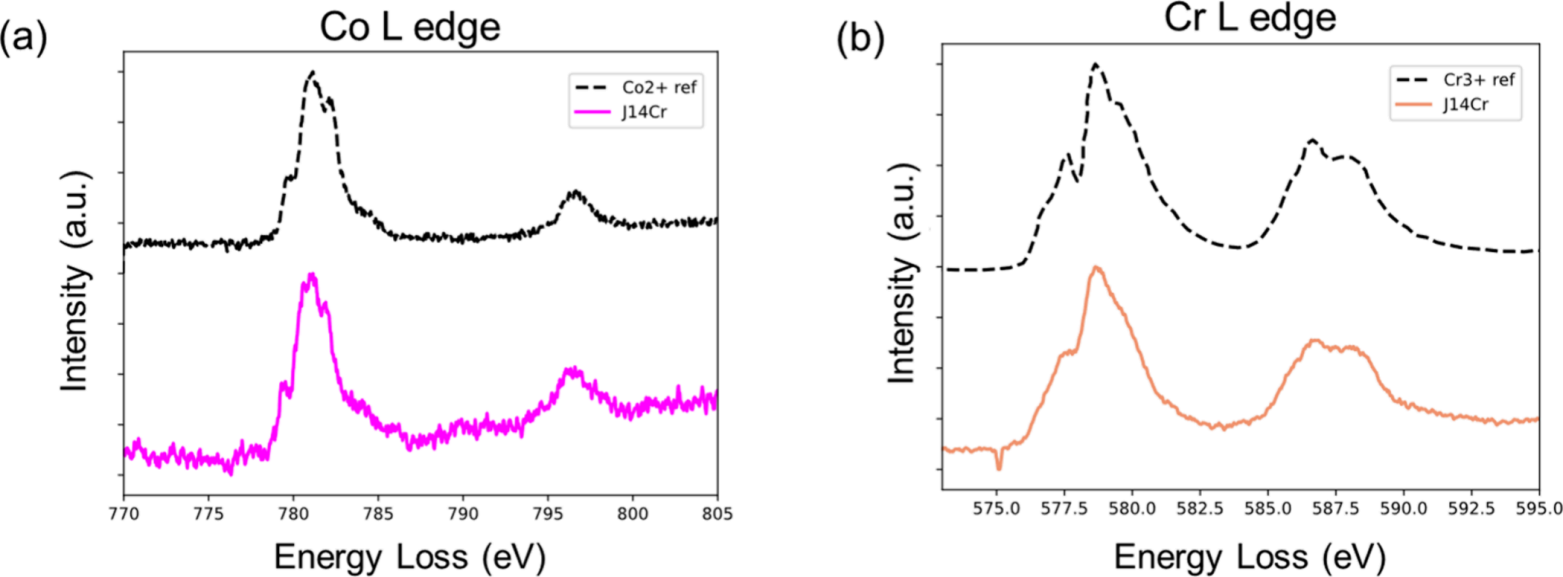
Investigation of oxidation state in J14Cr HEO: summed-up EELS data across the entire thin film of (a) Co L-edge and (b) Cr L-edge along with the references.^31,34^

To complement the EELS data, X-ray absorption spectroscopy (XAS) was performed. The results for the Mn, Cr, Co, and Cu K-edge from XAS are shown in the SI, Figure S6. According to these measurements, we independently confirm that the Mn K-edge energy of J14Mn (Figure S6a) is similar to that of Mn_3_O_4_, indicating an average oxidation state of around 2.667+. This is consistent with the mixed-valence state observed in EELS. The Cr K-edge of J14Cr is shown in Figure S6b. The edge energy value was determined by the position of the first inflection point in the spectra and then compared to the results found in the literature.^35^ This edge position shifts linearly with oxidation state and can therefore be used to estimate the oxidation state of an unknown sample. Based on the literature data,^35^ we determine that Cr in J14Cr occupies a primarily 3+ state, as the measured edge energy of Cr in J14Cr was about 9.93 eV above the Cr metal edge at 5989 eV. The XAS results for the Co K-edge from J14Mn and J14Cr are shown in Figure S6c. The shape and position of the Co K-edge of the J14Mn spectrum show a subtle shift to the right compared to that of J14Cr. While the Co K-edge of J14Cr closely matches the measured CoO standard, suggesting that Co remains in a consistent 2+ oxidation state, the subtle right shift in J14Mn indicates a mixed valence of 2+ and 3+, consistent with the EELS measurements. The Cu K-edge in both J14Cr and J14Mn (Figure S6d) reveals a 2+ valence state, consistent with EELS showing no variation in the Cu valence across the thin films (Figures S4 and S5).

Since certain cations in both thin films adopt higher valences than the 2+ state typically expected in a rock salt-type system such as MgO, we sought to understand how charge-neutrality is maintained. In these systems, charge-neutrality is believed to be achieved through cation deficiency, as observed in the parent J14.^6,7^ The atomic-resolution EELS chemical maps for J14Mn and J14Cr, presented in the SI (Figures S7 and S8, respectively), highlight regions exhibiting local cation redistribution (denoted by white circles), manifested as areas with relative cation enrichment and depletion, which we attribute to the charge compensation mechanism. Quantitatively, for J14Mn, Mg, Ni, Cu, and Zn have 2+ valence, while Co has an average of 2.2+ valence and Mn has an average of 2.9+ valence (based on EELS measurement as discussed in S12). This leads to average cation valence of 2.183+ in J14Mn. For charge-neutrality purposes, there should be ∼8.4% of cation vacancies in J14Mn. Similarly, for J14Cr, Mg, Co, Ni, Cu, and Zn have 2+ valence, while Cr is in the 3+ state, yielding an average cation valence of 2.167+. For charge-neutrality purposes, there should be ∼7.7% of cation vacancies in J14Cr.

## Discussion and Summary

Our S/TEM and EELS study elucidates that under the given growth conditions, J14Mn has cations occupying certain tetrahedral sites, leading to the coexistence of spinel and rock salt structures with cation vacancies for charge-neutrality, while J14Cr maintains a rock salt structure. Additionally, J14Mn has a smaller lattice parameter compared to J14Cr, which can be attributed to Mn and Co adopting higher oxidation states, thereby decreasing their ionic size and, consequently, reducing the overall unit cell volume.

To determine whether nanoscale spinels are observed in J14Mn at other thicknesses, we also investigated a thicker J14Mn thin film (∼110 nm), as shown in Figure S9. DF-TEM reveals that the spinel structure is randomly distributed throughout the thin film and at the interface, as shown in Figure S9b. A possible driving force for nanospinel formation could be the reduction of overall strain. We believe that the nanospinel formation is promoted by Mn^3+^and Co^3+^, which are comparable in size, thereby minimizing the overall strain. Previous studies have established that the coexistence of rock salt and spinel phases arises from substrate-induced strain, which governs the thermodynamic stability of these phases.^28^ Moreover, higher Mn concentrations in various HEO compositions have been reported to promote spinel formation.^27,36^ The coexistence of rock salt and spinel regions in HEOs has shown potential for applications as electrode material with tunable electrochemical behavior, enabled by optimizing the ratio between the two phases.^27^

To complement our observation of coexistence of rock salt and spinel regions, we calculated the cation-site-preference energy (CSPE) for all cation combinations in an AB_2_O_4_ spinel structure using density functional theory: CSPE = *E*_inverse_ – *E*_normal_. The relative tendencies for octahedral or tetrahedral coordination across relevant cation combinations can be quantified by this energy difference between normal (*E*_normal_) and inverse (*E*_inverse_) spinel structures. In normal spinels, A cations occupy tetrahedral sites and B cations occupy octahedral sites, while inverse spinels have A cations occupy 1/2 octahedral sites and B cations occupy tetrahedral sites and 1/2 octahedral sites. As shown in Figure 6, Cr has a strong preference for octahedral coordination for all explored cation combinations, providing support for the experimental observation of J14Cr being a rock salt structure. Comparatively, Mn has both an octahedral or tetrahedral site preference depending on the other cation, which could lead to some spinel-like structures. This understanding aligns well with our experimental analysis that J14Mn contains some coexistence of both rock salt and spinel.

**Figure 6:**
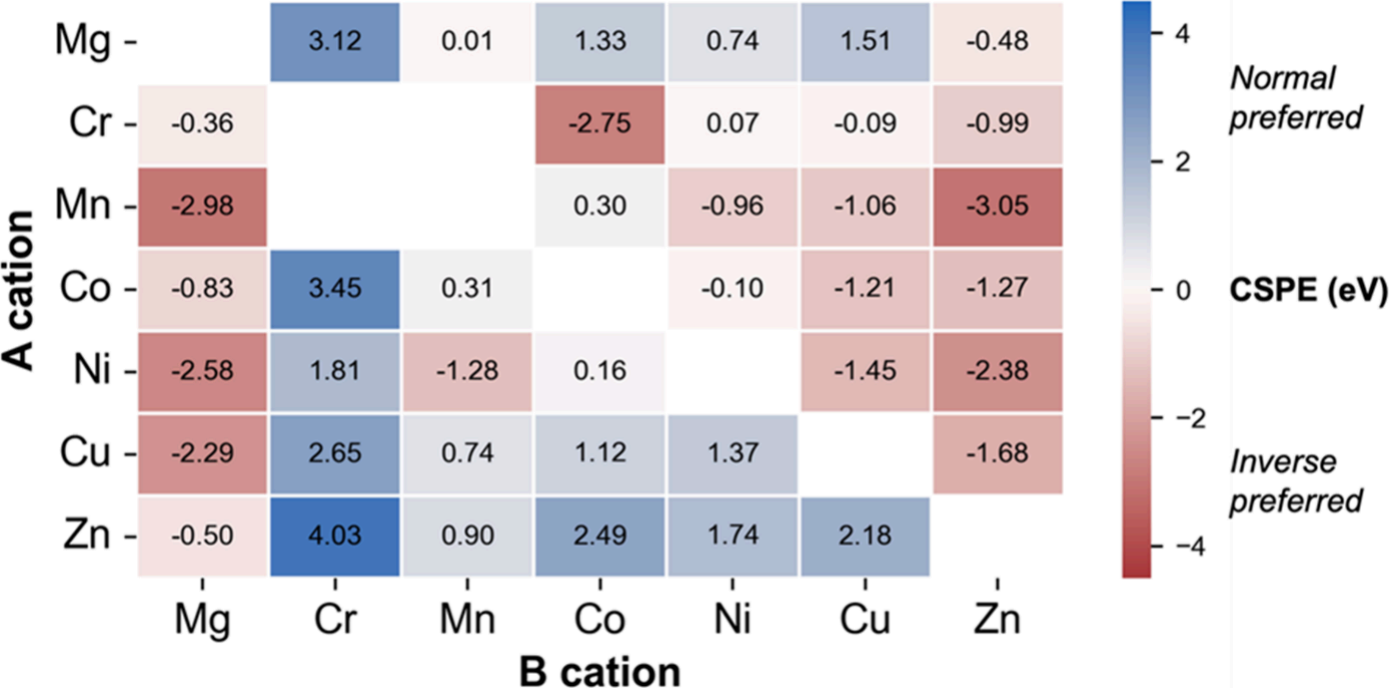
Cation-site-preference energies (CSPEs) for all AB_2_O_4_ spinel cation combinations calculated with density functional theory. Positive values (shown in blue) indicate preference for a normal spinel with A cations occupying tetrahedral sites and B cations occupying octahedral sites, while positive values (shown in red) indicate preference for an inverse spinel with B cations occupying tetrahedral sites and half of the octahedral sites with A cations occupying the remaining half of the octahedral sites. Cation combinations containing both Cr and Mn have been excluded for clarity across explored J14Cr and J14Mn compositions.

The Mn and Co valence shifts to mixed 2+ and 3+ states, combined with cation deficiency, drive some cations to tetrahedral sites, forming spinel-like regions within the rock salt matrix.

Furthermore, the spinel-like regions in J14Mn do not have a well-defined shape; rather, they are coherently intermixed with the rock salt matrix without any interfacial boundary. This differs from the well-defined spinel cuboids previously observed in the J14 system, which could be attributed to differences in interfacial energy due to the presence of Mn and Co ions in J14Mn.^8^ Interestingly, we note that Co exhibits CSPE values between those of Cr and Mn for J14 possible cation combinations, aligning with our ability to coerce J14 into both rock salt-containing nanospinel regions^8^ as well as a rock salt with significant cation vacancies.^6,7^ As PLD enables us to quench these metastable states in J14, we hypothesize that unique metastable nanostructures can be captured in J14Cr and J14Mn by altering these nonequilibrium growth conditions.

The larger exchange bias observed in J14Mn indicates stronger pinning of magnetic moments, i.e., increased spin frustration at the ferromagnet and antiferromagnet interface, whereas J14Cr exhibits almost no exchange bias, suggesting more glassiness (disorder) in magnetic moments. This higher exchange bias of J14Mn can be hypothesized to originate from the presence of spinel-like nanoregions and local variations in valences of Co ions. We propose that the transition of Co^2+^ (a d^7^ magnetic ion) in the rock salt to some Co^3+^ (a d^6^ magnetically inert ion) in spinel-like regions results in uncompensated spins and magnetic dilution, leading to an enhanced exchange bias.

We have attempted to measure a magnetic moment from these films but were not able to detect a measurable signal. This suggests that the total volume fraction of spinel regions is too small for a VSM measurement. Prior work by Meisenheimer et al.^21^ and Kotsonis et al.^6^ has shown that Co^3+^in the rock salt structure dilutes the density of magnetic species and leads to increased magnetic frustration as it is in a low-spin configuration without an atomic moment. We note that the spinel precipitates are a few unit cells in size and therefore unlikely able to support coherent ferrimagnetism but are likely to yield uncompensated magnetic moments that can influence the exchange bias and the bulk magnetic anisotropy of the film. The exchange bias effect is dominantly interfacial with a contribution of the bulk magnetic anisotropy of the HEO that extends from the interface by the exchange length (∼50–100 nm). Given the compositional and valence changes due to the precipitates (both the spinel phase and low-spin Co) and their relatively significant population, we anticipate enhanced magnetic frustration in the system. This expectation is consistent with the broadened Py hysteresis loop and no clear presence of magnetic anisotropy.

Further experiments like temperature-dependent magnetization studies would help clarify the nature of the antiferromagnetic behavior. Previous studies have reported that HEOs exhibit a high degree of magnetic frustration, which can be influenced by varying Cu concentration^21^ or by altering the growth kinetics.^6^ Additionally, J14Mn shows a higher Néel temperature compared to J14Cr, which we attribute to the presence of increased defect states in the system, consistent with observations in parent oxides such as NiO and MnO.^37^

In conclusion, we have demonstrated that the introduction of Mn to J14 results in the coexistence of rock salt and spinel-like regions, along with valence changes of Co and Mn ions, whereas the introduction of Cr to J14 results in a rock salt structure. We attribute the charge compensation in these systems primarily to cation vacancies. Our findings highlight that subtle nuances in local structure, chemistry, local strain energies, and cation valences strongly influence the magnetic behavior of these compositionally complex systems. This study further emphasizes that high-entropy oxide materials offer a vast compositional space, with design flexibility and tunable properties enabled by careful control of local structure and chemistry.

## Experimental Section/Methods

### Material Synthesis

(Mg_0.167_Co_0.167_Ni_0.167_Cu_0.167_Zn_0.167_Mn_0.167_)O and (Mg_0.167_Co_0.167_Ni_0.167_Cu_0.167_Zn_0.167_Cr_0.167_)O were synthesized by mixing the binary oxide components at equimolar stoichiometry, milled and pressed into pellets. The high-entropy oxide thin films of target compositions were grown on the [001] oriented MgO substrates using pulsed laser deposition. The target-substrate distance was 7 cm and a 248 nm KrF laser was fired at a pulse rate of 5 Hz with laser fluence of 1.7 J cm^–2^ with a spot size of around 0.05 cm^2^ for a total of 6000 pulses. The substrates were baked at 950 °C in base vacuum for 20 min prior to being held at 400 °C in 5 mTorr of pure O_2_ throughout the deposition before cooling at the same pressure.

### Material Characterization

#### X-ray Diffraction

Following the deposition, 2θ-ω X-ray diffraction scans were performed with a Rigaku Smartlab diffractometer at the University of Michigan, using a 1.54 Å Cu Kα source, a Ge 220 monochromator, and parallel beam geometry. A 5 mm slit was used, with a 0.1-degree step size; for narrow scans, the speed was 1 degree per minute, while for broad scans, the speed was 12 degrees per minute.

#### Exchange Bias Magnetic Measurements

Magnetometry measurements were made in a Quantum Design PPMS with a vibrating sample magnetometer attachment. Measurements were made as a function of temperature spanning from 300 to 5 K. Moment versus temperature measurements were made upon heating after 50 Oe (ZFC) and 2000 Oe field cooling (FC). Magnetic hysteresis loops were taken at temperatures below and above the blocking temperature to evaluate the loop of the permalloy layer and the role of the underlying HEO film. Loops were fitted to a Stoner-Wolfarth model with interface exchange coupling and disorder for quantifying the degree of spin ordering and exchange bias.

#### Sample Prep for S/TEM Studies

Sample preparation for S/TEM studies was carried out using an FEI Helios 660 Dual Beam focused ion beam (FIB). The TEM lamellae were extracted and thinned at 30 and 5 kV ion beam, respectively. The final cleaning was performed at 2 and 1 kV ion beam to minimize the damage caused by Ga^+^ ions. Multiple FIB samples were made from different locations and investigated to ensure that the local structural variation is not induced by FIB.

The J14Mn lamella used to perform the atomic-resolution EELS experiments, shown in Figure 3, was prepared using Hitachi Ethos NX500 Ga^+^ ion FIB at SuperSTEM, UK, also using progressively lower beam acceleration voltages, from 30 to 2 kV. The lamella was polished at low kV using the coincident collimated Ar ion beam of the triple-beam Hitachi Ethos.

#### Scanning/Transmission Electron Microscopy (S/TEM)

An FEI Talos 200X2 operated at 200 kV accelerating voltage was used to perform selected area electron diffraction and dark-field TEM experiments. Low-magnification STEM-EELS experiments were carried out on a double Cs corrected FEI Titan G2 equipped with a monochromator at an accelerating voltage of 300 kV.

##### Unsupervised Machine Learning on STEM Images

In-house-developed Python code was used to perform the unsupervised machine learning clustering on the STEM images. The HAADF-STEM images were loaded using Hyperspy,^38^ and a sliding window of 64 by 64 pixels was moved across the STEM image with a step size of 4 pixels. Different sliding window sizes and step sizes were tested, and the results remained consistent. For each window, the FFT was computed and squared to obtain the power spectrum |FFT|^2^, and principal component analysis (PCA) was applied to the standardized data by using the scikit-learn implementation. The number of clusters was estimated from the elbow point in the PCA scree plot (Figure S1c,e). To perform the unsupervised clustering, a Gaussian mixture model (GMM) was applied to the PCA-transformed data.

##### Virtual Dark-Field STEM Imaging using 4D STEM

The 4D STEM data were acquired on a Nion UltraSTEM 100 at Oak Ridge National Laboratory using a Dectris detector operating at 1000 frames per second. Experiments were carried out at an accelerating voltage of 100 kV with a probe convergence angle of 2.5 mrad to avoid overlapping of CBED disks. In real space, the probe step size was 0.5 nm. The 4D STEM data were processed using the py4DSTEM package in Python.^39^

##### STEM Image Simulations

STEM image simulations were executed using the open-source abTEM package in Python.^40^ The simulation parameters were chosen to closely match the experimental conditions. A C_s_ of 0 nm, annular detector with a collection angle of 90 to 190 mrad, and 10 frozen phonon configurations with 0.1 Å standard deviation in atomic displacements to sample different thermal configurations were used to perform stem image simulations. A modified crystal structure of Co_3_O_4_ with lattice parameter of 8.56 nm was used to simulate the spinel structure, while MgO with lattice parameter of 4.28 nm was used to simulate the rock salt structure.

##### Electron Energy Loss Spectroscopy

The EELS data sets in Figure 5 and Figure S3 and S4 are acquired on Titan^3^ G2 at 300 kV with a monochromated beam with 0.2 eV full width half max (fwhm) of zero-loss peak (ZLP) at the smallest dispersion at Penn State. Atomic-resolution STEM-EELS measurements were performed on aberration-corrected Nion UltraSTEM100MC-Hermes at 60 kV accelerating voltage at SuperSTEM, UK. This instrument has a cold field emission electron source with a native 0.3 eV fwhm of the ZLP, and the spectra analyzed here were acquired using an energy dispersion of 0.1 eV per channel. A beam with a semiangle convergence of 30 mrad (and a probe current of 35pA, for an estimated probe size of 0.1 nm) and collection semiangle of 44 mrad was chosen for atomic-resolution EELS maps. The microscope was equipped with a Nion IRIS spectrometer and a Dectris ELA camera for EELS.

Atomic-resolution HAADF intensity was recorded simultaneously with the maps on a detector with a 92–185 mrad semiangular range. EELS spectrum images were acquired as multipass consecutive data sets with fast dwell times per pixel (5 ms), before rigid registration and averaging. DigitalMicrograph software (DM) was used to process EELS chemical maps, while HyperSpy was used to analyze the EELS spectra.^38^

To process the chemical maps, the data sets were first denoised in DM using the “simple denoise”, retaining the first 30 principal components. A power law function was used to fit and subtract the pre-edge background, and the Hartree-Slater cross-section model was used for obtaining the maps. For J14Mn chemical maps, the O K-edge was fit over 512.7 to 635.4 eV, Mn L-edge was fit over 606.8 to 773.2 eV, while the Co L-edge, Ni L-edge, Co L-edge, Cu L-edge, and Zn L-edge were all fit over 701.7 to 1297.5 eV, and the Mg K-edge was fit over 1135.4 to 1364.8 eV. For J14Cr chemical maps, the O K-edge and Cr L-edge were fit over 509.0 to 776.4 eV, while the Co L-edge, Ni L-edge, Co L-edge, Cu L-edge, and Zn L-edge were all fit over 634.2 to 1299.2 eV, and the Mg K-edge was fit over 1082.9 to 1381.7 eV.

To quantify the valences of Co and Mn in the J14Mn thin film, we performed L_3_/L_2_ ratio and compared it with the existing literature.^32^ We utilized digital micrograph to perform the L_3_/L_2_ ratio, where a stepped continuum function under the EELS white lines, using a double-arctangent function in digital micrograph was used (Figure S5). In the literature, a step function of 2:1 is used for the background treatment. We performed the L_3_/L_2_ ratio on our experimental data to calculate the average oxidation states of Co and Mn in the J14Mn thin film with similar step background treatment as the literature (S12).

The Co L-edge references are from CoO and Co_3_O_4_.^31^ The CoO reference is obtained at 200 kV incident beam energy with 0.3 eV energy resolution, 0.05 eV/pixel dispersion, and a semiconvergence angle of 8 mrad. The Co_3_O_4_ reference is acquired at 200 kV incident beam with 0.35 eV resolution, 0.05 eV/pixel dispersion, and a semiconvergence angle of 3 mrad. Since the fine structure of EELS is dominated by energy resolution and both our reference data and experiments (Figure 3) have similar energy resolutions of 0.3 eV, they are comparable.

The Cr L-edge reference is from an X-ray absorption spectroscopy (XAS) experiment.^34^ EELS and XAS are complementary techniques that show similar fine structures because both probe the same unoccupied electronic states via core-level transitions to the same final states (3d bands for L-edges).

The Mn L-edge references are acquired at 200 kV accelerating voltage with an energy resolution of 0.25 eV and an energy dispersion of 0.05 eV/channel with a semiconvergence angle of 1.9 mrad.^30^ Since our experimental data (Figure 3) are acquired at 0.3 eV resolution, the EELS spectrum obtained experimentally is slightly broader than the reference data.

#### X-ray Absorption Spectroscopy

X-ray absorption fine structure (XAFS) measurements of the HEO thin films were performed at beamline 10-BM at Argonne National Laboratory (Lemont, IL) and collected in fluorescence mode with a Lytle detector. The powder standards used to compare valence state were measured on an EasyXAFS 300+ (Renton, Wa)^41^ operating an Ag X-ray tube at 35 kV and 25 mA and collected in transmission mode with a silicon drift detector. Data from both sources were measured with a reference metal foil used to calibrate absorption edge energy. The XAFS data were then processed and analyzed using the Demeter package for XAS analysis.^42^

#### Density Functional Theory Calculations

The Vienna Ab-initio Software Package (VASP) 6.4.1 is used for DFT calculations with the projector augmented-wave pseudopotentials v54.^43^ The regularized-restored strongly constrained and appropriately normed (r^2^SCAN) functional is used for its improved accuracy for transition-metal oxide systems.^44,45^ We restrict our calculations to the primitive spinel unit cell containing two formula units (2 tetrahedral cations, 4 octahedral cations, and 8 oxygen), which has been shown to only contain a single symmetrically unique distinct inverse spinel cation decoration and is capable of resolving stability differences between normal and inverse spinels.^46^ A k-point mesh of 6 × 6 × 6 centered on Gamma was used, with all other calculation parameters being unchanged from the Materials Project MPScanRelaxSet for r^2^SCAN calculations.^47,48^ The Pymatgen^49^ and Custodian^49^ packages were used to set up and manage calculation workflows.

## Supplementary Material



## Notes

Additional experimental results, including XRD patterns, virtual dark-field images from 4D STEM, XAS spectra, EELS data with analysis, and chemical maps supporting the main text (PDF)

## Abbreviations

HEO: High-entropy oxide
STEM: Scanning transmission electron microscopy
EELS: Electron energy loss spectroscopy
HAADF: High-angle annular dark-field
XRD: X-ray diffraction
EDS: Energy-dispersive spectroscopy
FFT: Fast Fourier transform
PLD: Pulsed laser deposition
