# Supplementary material for: Evidence of Local Structural Variations and Their Influence on Magnetic Properties in Mn- and Cr-Containing High-Entropy Oxide Thin Films Using Electron Microscopy
Source: J Am Chem Soc. 2026 May 28;148(22):22521–31. doi: 10.1021/jacs.6c00090 (PMC13266977; doi:10.1021/jacs.6c00090)
Supplement: Supplementary file 1 [file ja6c00090_si_001.pdf]

SUPPLEMENTAL INFORMATION:

Evidence of Local Structural Variations and Their Influence on Magnetic Properties in Mn- & Cr-Containing High Entropy Oxide Thin Films Using Electron Microscopy

Sai Venkata Gayathri Ayyagari[1], Matthew Webb[2], Jacob T. Sivak[3], Gerald Bejger[4], John P. Barber[4], Debangshu Mukherjee[5], Kevin M Roccapriore[6,7], Aleksander B. Mosberg[8], Leixin Miao[1], Jon-Paul Maria[1], Susan B. Sinnott[1,3,9], Christina M. Rost[4], Quentin M. Ramasse[8,10], John T. Heron[2], Nasim Alem[1*]

1. Department of Materials Science and Engineering, The Pennsylvania State University, University Park, Pennsylvania, 16802, USA.
2. Department of Materials Science and Engineering, University of Michigan, Ann Arbor, Michigan, 48109, USA.
3. Department of Chemistry, The Pennsylvania State University, University Park, Pennsylvania, 16802, USA.
4. Department of Materials Science and Engineering, Virginia Polytechnic Institute and State University, Blacksburg, Virginia, 24061, USA.
5. Computational Sciences & Engineering Division, Oak Ridge National Laboratory, Oak Ridge, Tennessee, 37830, USA.
6. Center for Nanophase Materials Sciences, Oak Ridge National Laboratory, Oak Ridge, Tennessee, 37830, USA.
7. AtomQ, Knoxville, Tennessee, 37931, USA.
8. SuperSTEM Laboratory, SciTech Daresbury Campus, Daresbury, WA4 4AD, UK.
9. Institute for Computational and Data Sciences, The Pennsylvania State University, University Park, Pennsylvania, 16802, USA.
10. School of Chemical and Process Engineering & School of Physics and Astronomy, University of Leeds, Leeds, LS2 9JT, UK.

S1: XRD PATTERNS AND UNSUPERVISED ML ANALYSIS OF STEM IMAGE:

Figure S1 shows the XRD plots and additional results from the unsupervised ML analysis on STEM images. The principal component analysis (PCA) scree plot illustrates the variance within the dataset, and the elbow point is typically used to determine the number of components present. The red arrow in Figure S1c and Figure S1e indicate the number of components used in the analysis. The FFT differences between clusters are shown in Figure S1d and Figure S1f for J14Mn and J14Cr, respectively.

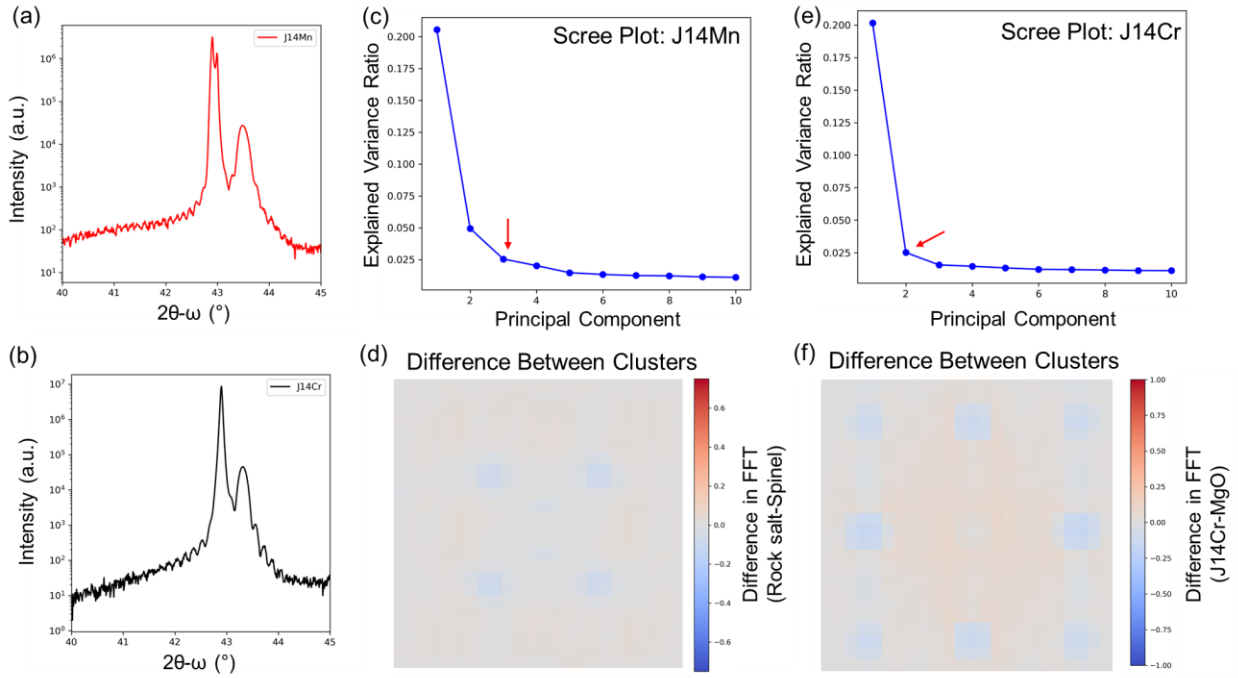

Figure S1: (a) X-ray diffractograms centered on the (200) reflection of the MgO substrate for J14Mn and (b) J14Cr. (c) Scree plot of J14Mn with a red arrow indicating the elbow point used to determine the number of clusters. (d) Difference in average FFT of rock salt and spinel regions, showing that the extra reflections originate from the spinel region. (e) Scree plot of J14Cr. (f) Difference in average FFT of J14Cr and MgO, showing no evident variation in lattice parameter.

S2: VIRTUAL DARK FIELD IMAGING USING 4D STEM:

Figure S2 shows the 4D STEM results. We note a relative rotation of CBED patterns with respect to the sample orientation.

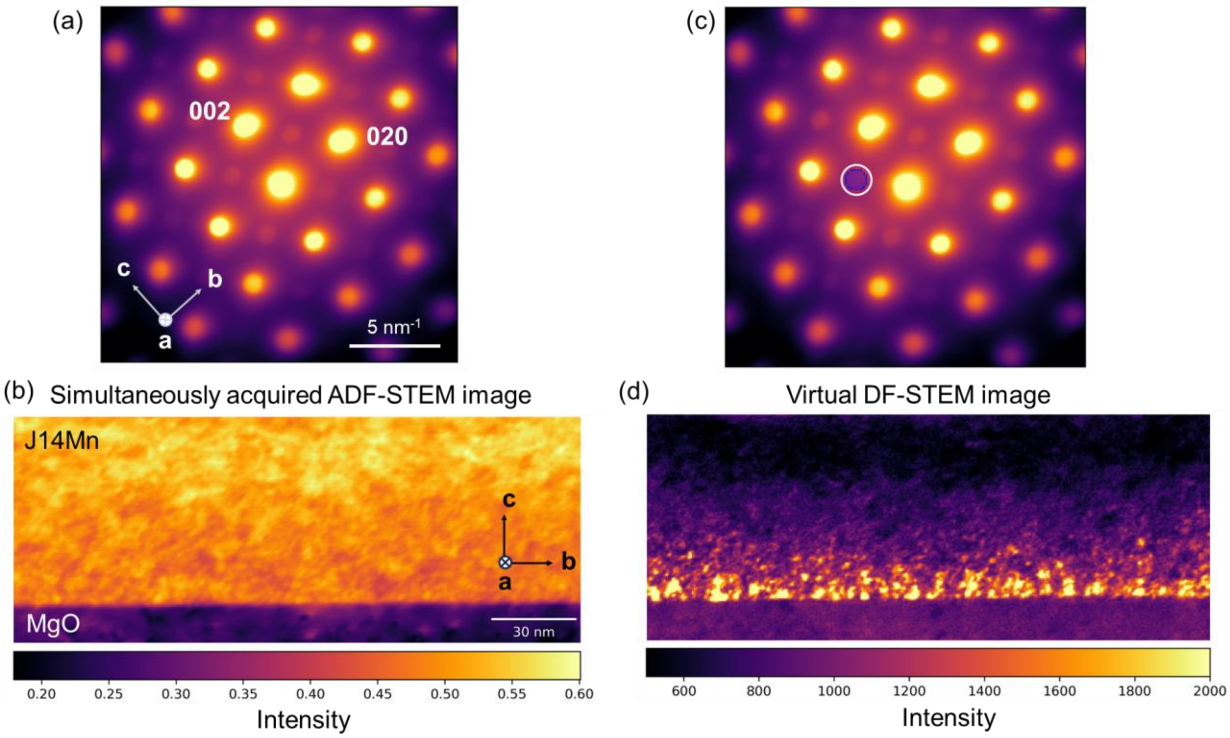

Figure S2: (a) Mean CBED dataset across the entire 4D STEM dataset. (b) Simultaneously acquired ADF-STEM image showing the region of 4D STEM data. (c) Mean CBED data (same as (a)) with the inset showing the reflection used to acquire the virtual dark field STEM image shown in (d).

S3 AND S4: OXIDATION STATE INVESTIGATION ACROSS THIN FILMS:

Figure S3 and S4 shows the monochromated EELS across the thin films for J14Mn and J14Cr, respectively. First, an EELS scan ranging from 470 eV to 980 eV is performed across the entire cross-section of the thin film. The colors used for the EELS edges correspond to the colors of the probe positions, and the line scan was taken along a slanted trajectory to sample a larger area of the thin film. This energy range allows simultaneous probing of the O K edge, Cr L edge, Mn L edge, Co L edge, Ni L edge, and Cu L edge. The other cations in the J14Mn and J14Cr system, Zn and Mg, are always present in the 2+ state, so they are not analyzed. In this analysis, the core loss is aligned with respect to the zero-loss peak, and every three datasets were summed to enhance the signal to noise ratio. Ni L edge does not change, and the peak shape matches with 2+ valence for both J14Mn and J14Cr thin films.

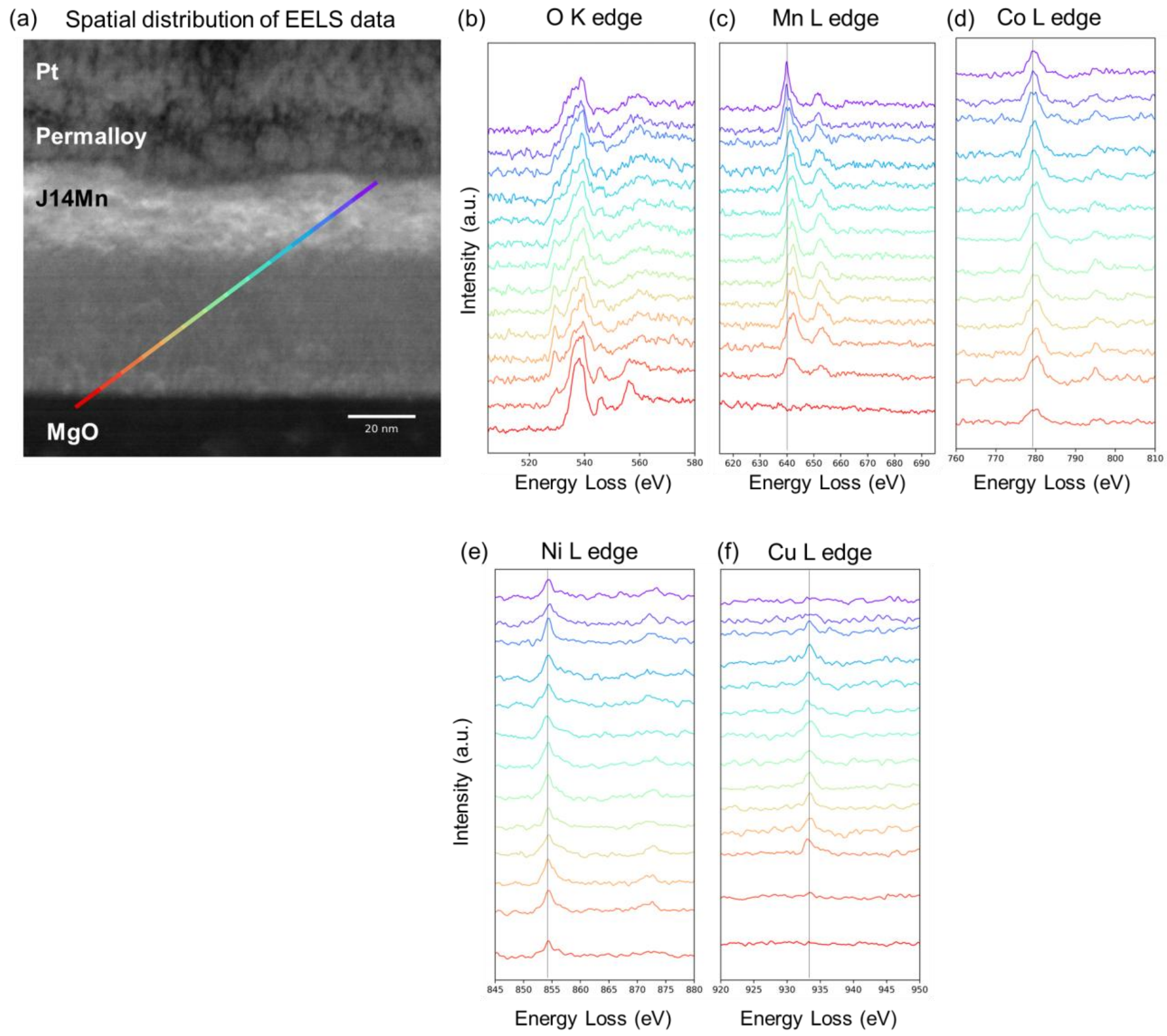

Figure S3: (a) STEM image of J14Mn thin film, with each color corresponding to regions from which EELS spectra were summed and plotted in (b-f). (b-f) Spectra corresponding to the O K edge, Mn L edge, Co L edge, Ni L edge, and Cu L edges, respectively.

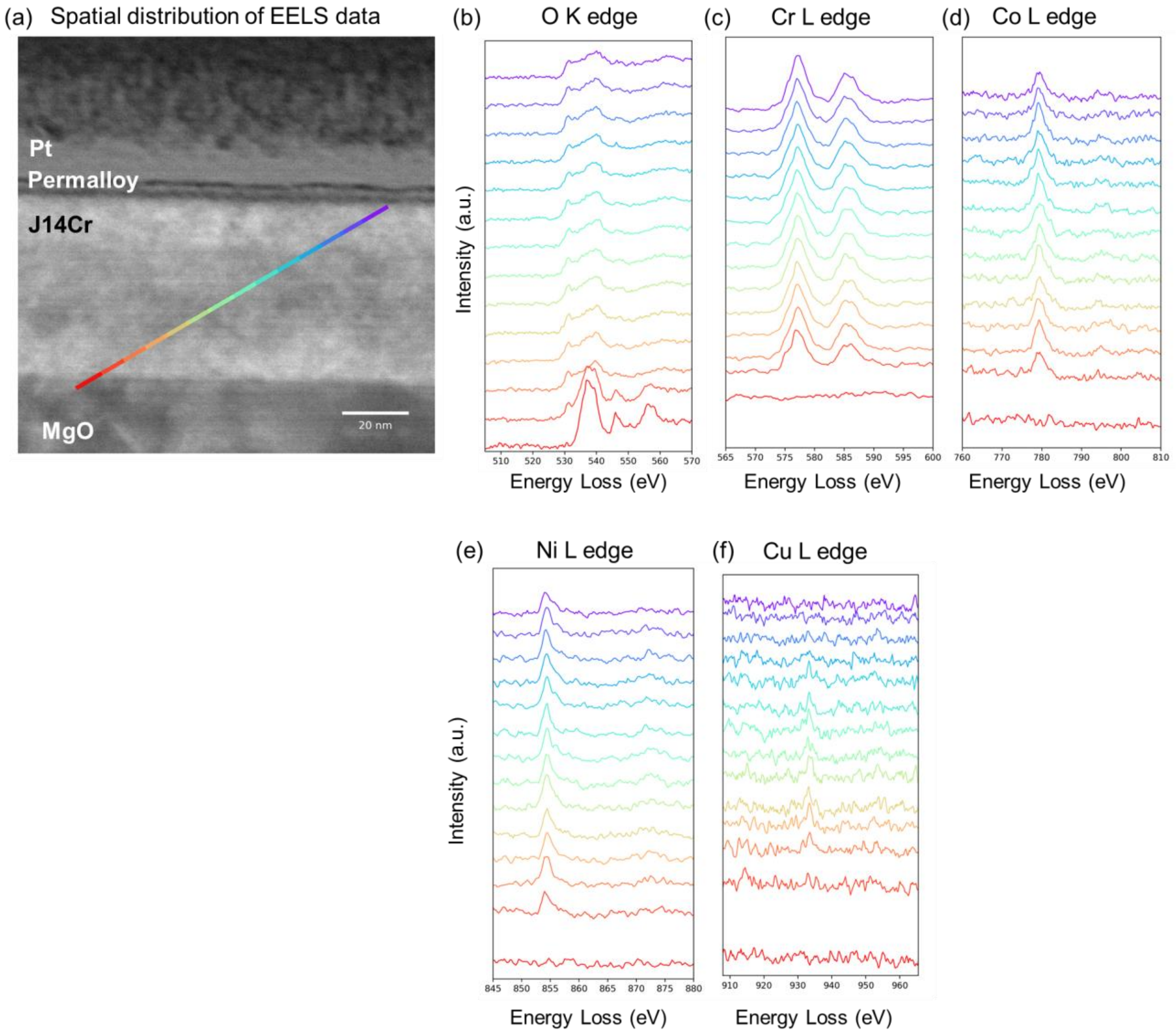

Figure S4: (a) STEM image of J14Cr thin film, with each color corresponding to regions from which EELS spectra were summed and plotted in (b-f). (b-f) Spectra corresponding to the O K edge, Cr L edge, Co L edge, Ni L edge, and Cu L edge, respectively.

S5: EELS L_3 BY L_2 RATIO CALCULATION:

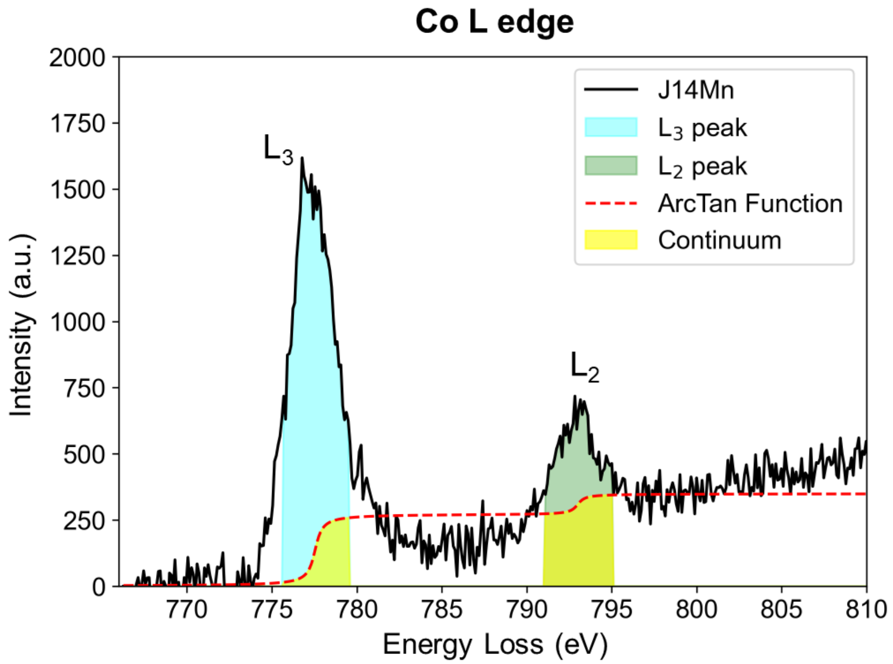

Figure S5: Co L_3/L_2 calculation on the rock salt Co L edge in J14Mn (shown in Figure 3a) by creating a stepped continuum function under the EELS white lines, using a double arctangent function in digital micrograph.

S6: X-RAY ABSORPTION SPECTROSCOPY:

Figure S6 represents the X-ray absorption spectroscopy results of Mn, Cr, Co, and Cu K edges in J14Mn and J14Cr thin films. These spectra are compared with the measured standards to determine the oxidation states.

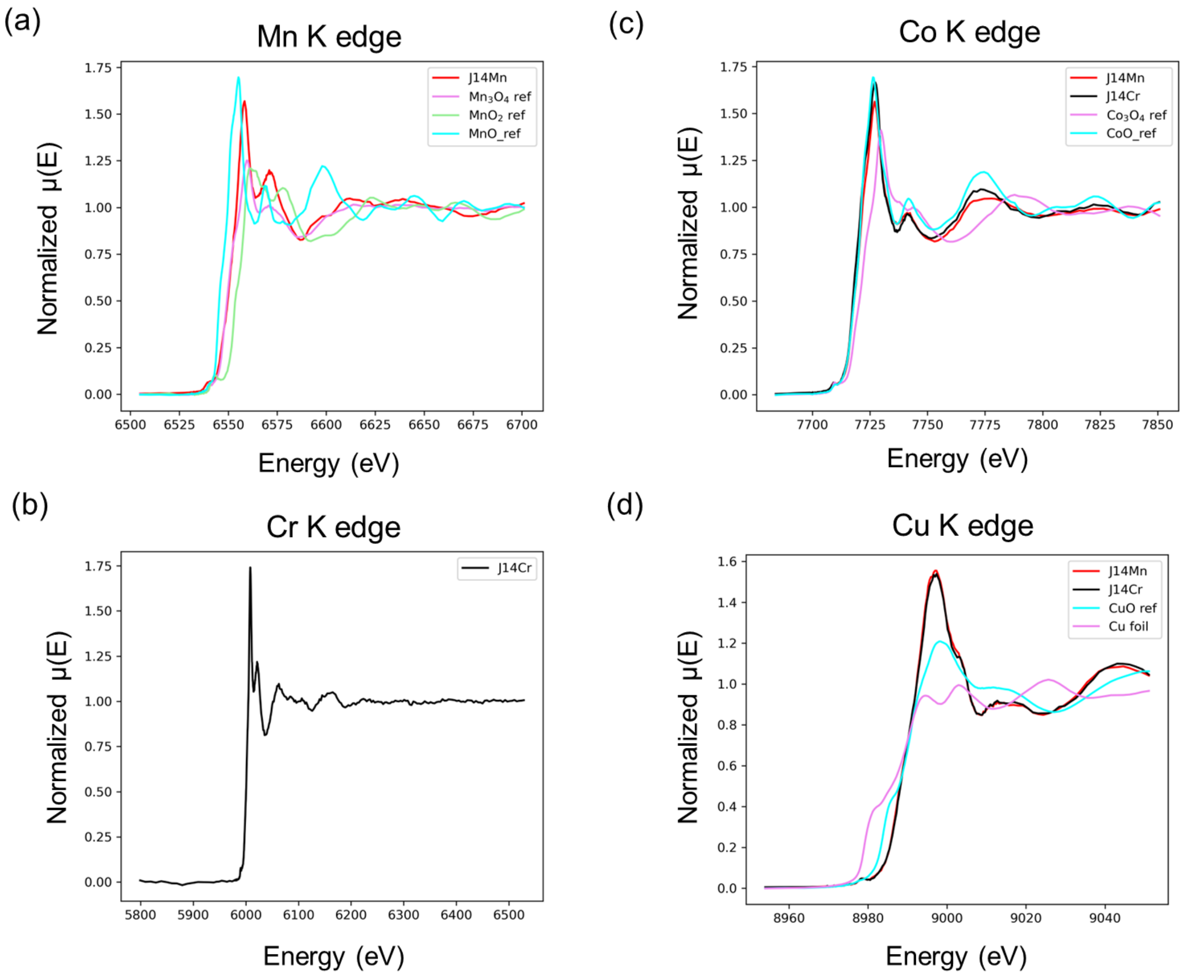

Figure S6: X-ray absorption spectroscopy of (a) the Mn K edge from J14Mn overlaid with Mn_3O_4, MnO, and MnO_2 standards. (b) The Cr K edge from J14Cr. (c) The Co K edge from J14Mn and J14Cr overlaid with Co_3O_4 and CoO standards. (d) The Cu K edge from J14Mn and J14Cr overlaid with CuO and Cu foil standards.

S7 AND S8: COMPOSITION MAPS FROM EELS:

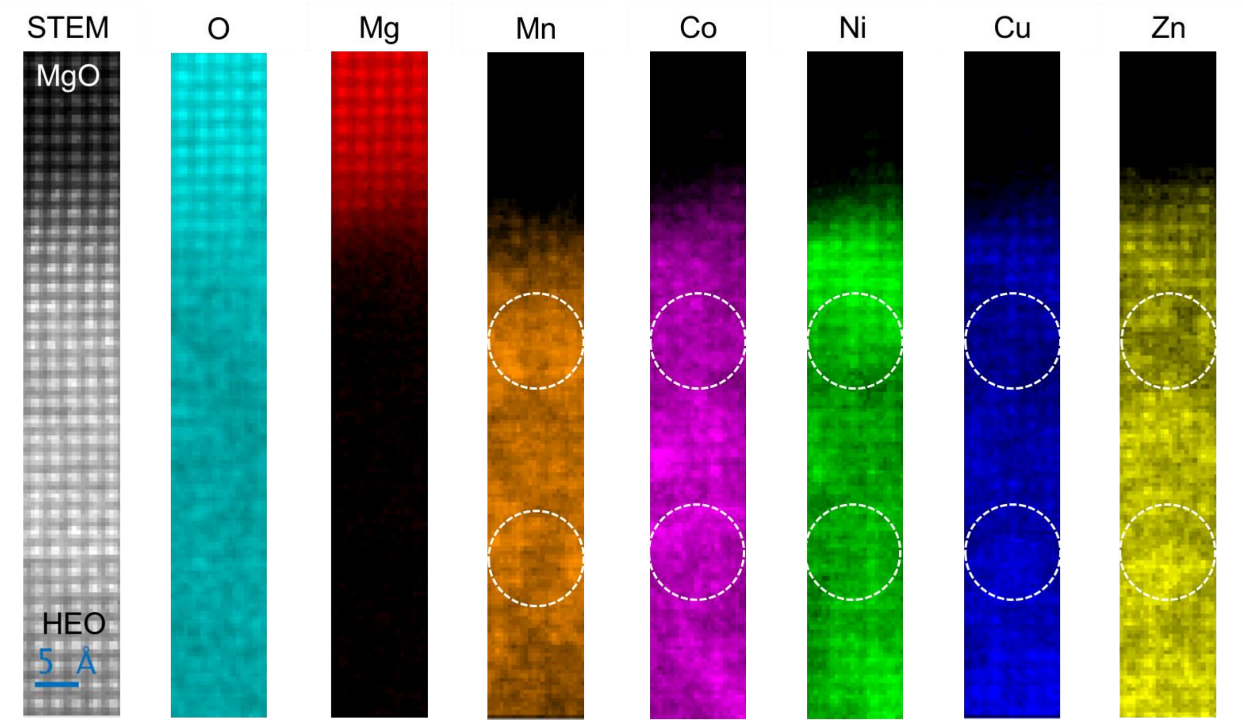

Figure S7: EELS chemical composition maps of the J14Mn thin film, with white insets highlighting local variations in chemistry.

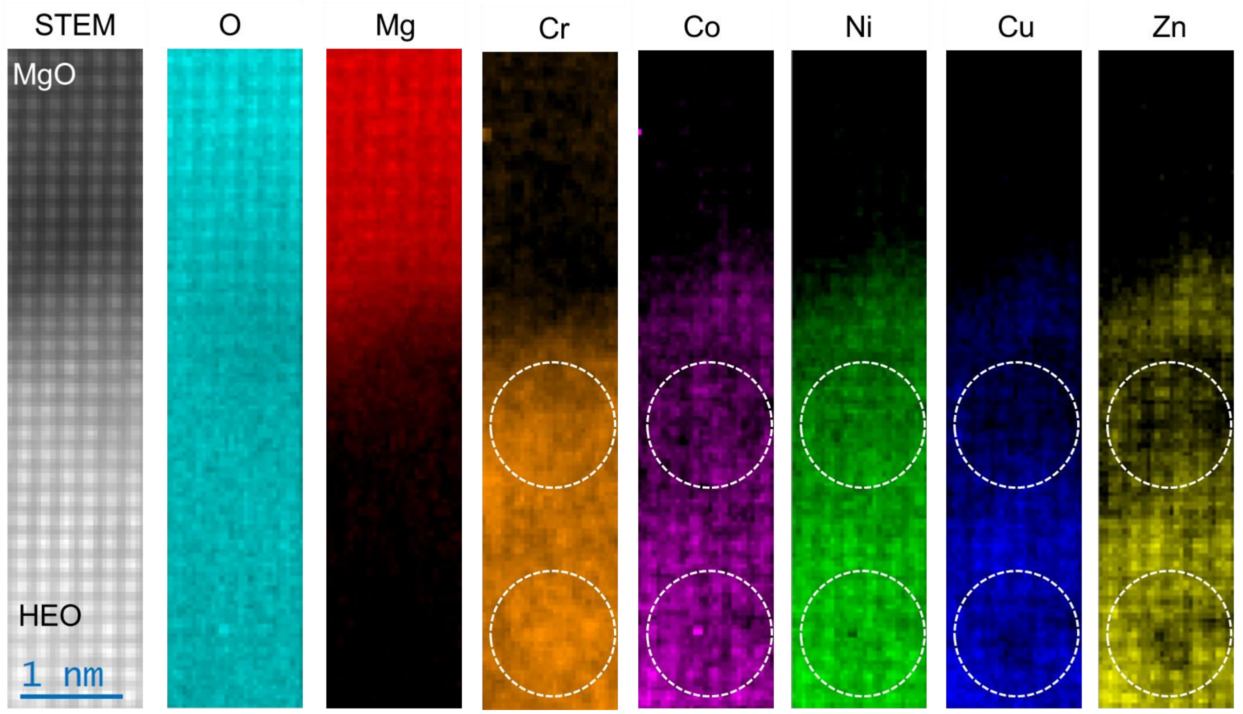

Figure S8: EELS chemical composition maps of the J14Cr thin film, with white insets highlighting local variations in chemistry.

S9: STRUCTURAL INVESTIGATION ON J14MN THIN FILM ~ 110 NM THICKNESS WITH NO PERMALLOY OR PT LAYERS ON THE TOP OF THIN FILM:

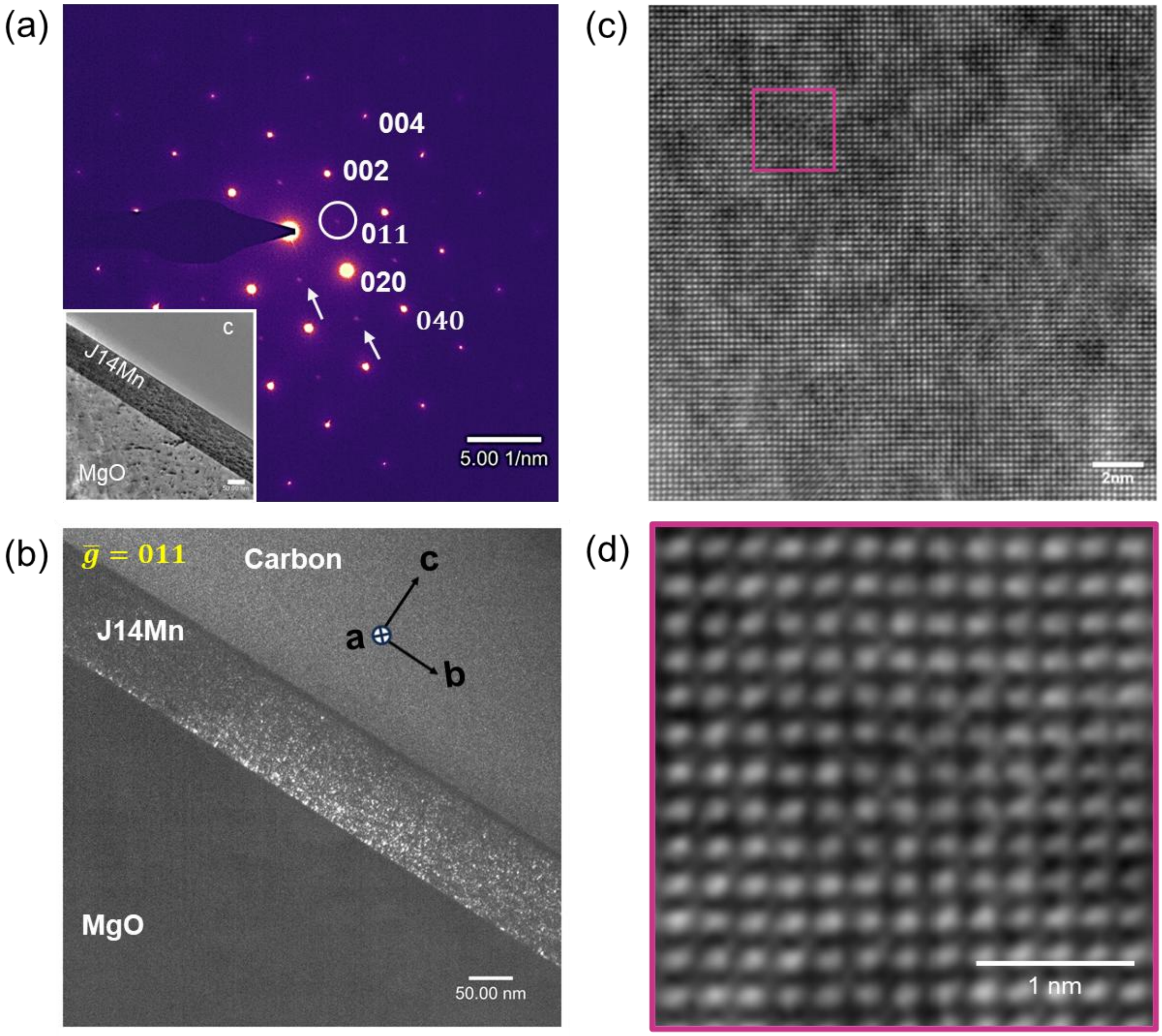

Figure S9: (a) Selected-area electron diffraction pattern of the J14Mn thin film (~110 nm thick) along with the corresponding selected-area region (scale bar: 50 nm), with arrows pointing to extra reflections that are typically absent in the rock salt structure along [100] zone axis. The circular inset corresponds to the DF-TEM image shown in (b). (c) Atomic resolution HAADF-STEM image of the J14Mn, with a magnified view of spinel-like region shown in (d).

S10: ELEMENTAL DISTRIBUTION MAPS OF J14MN THIN FILM

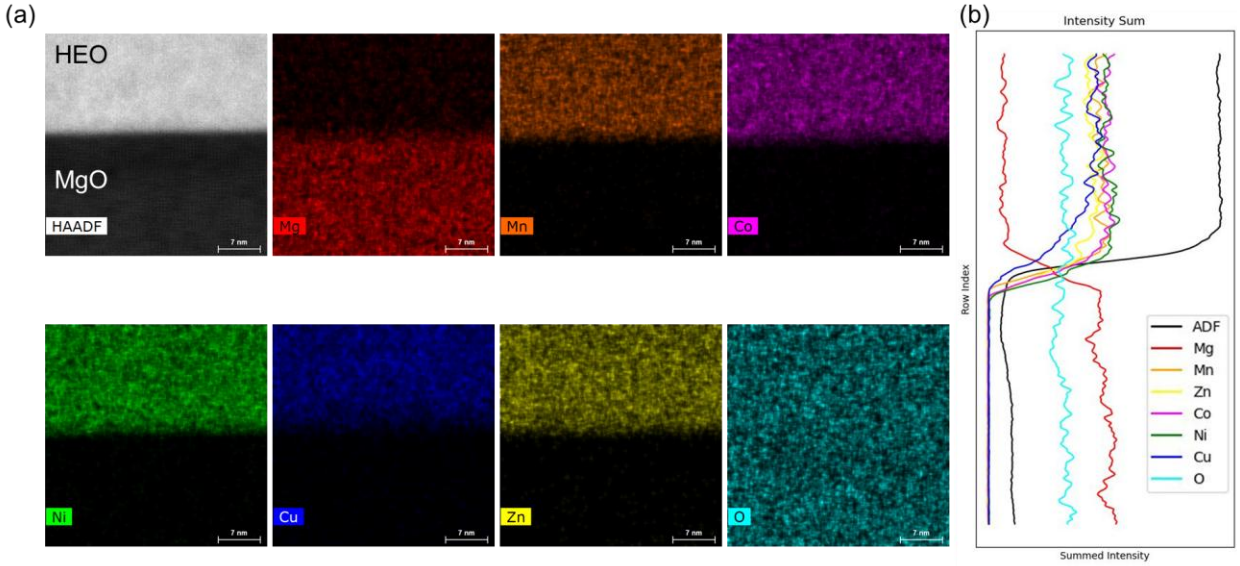

Figure S10: (a) EDS elemental maps of J14Mn thin film at the thin film/substrate interface. (b) Row-summed intensity profiles from EDS maps. No evidence of Mg enrichment at the interface.

S11: COMPARING THE OXYGEN CONTENT IN J14MN VS. J14CR THIN FILMS:

The integrated intensity ratio of O K-edge with respect to Ni L-edge is calculated. Ni was chosen as a reference because it does not change valence across both the J14Mn and J14Cr thin films. This ratio normalizes the effect of experimental conditions such as thickness variation, electron count, etc. According to this calculation, the O K / Ni L ratios are ~1.60 for J14Mn and ~1.52 for J14Cr, confirming no significant difference in oxygen content. Thus, spinel emergence in J14Mn reflects Mn-driven cation redistribution/vacancies, independent of O content.

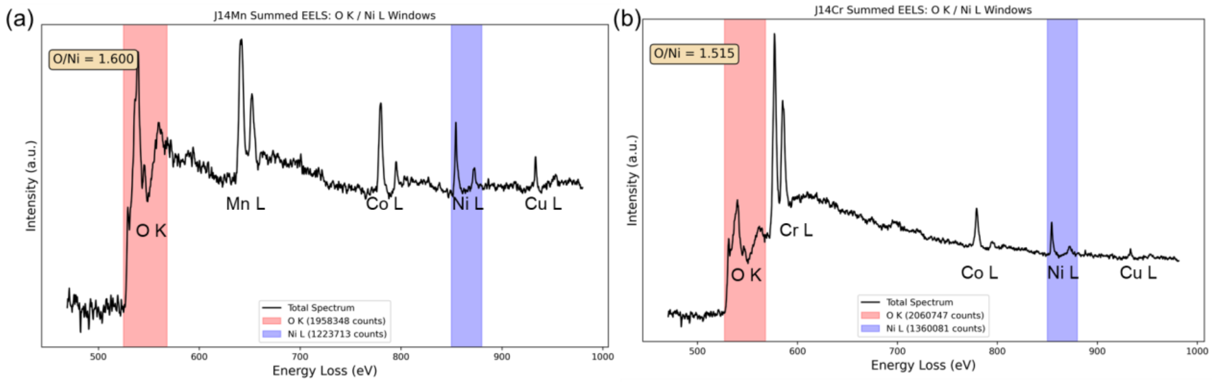

Figure S11: (a-b) Average EELS data of J14Mn and J14Cr thin film, respectively. The integrated ratio of O K edge with respect to Ni L edge is calculated. The window of O K edge is 527 – 568 eV and Ni L edge is 850 – 880 eV.

S12: CALCULATION OF CATION DEFICIENCY USING AVERAGE VALENCE OF CATIONS FROM EELS:

The average valence of Co and Mn in J14Mn is calculated across the entire thin film. We sum up the entire EELS data acquired on the thin film in Figure S3. The L_3/L_2 ratio is calculated by using a step background function to be consistent with the reference[1]. We perform similar background treatment on the reference dataset to ensure accuracy of the background step model.
In case of Co L edge,
CoO reference[2] $L_3/L_2 = \sim 4.6$,
average J14Mn EELS data (Figure S3) $L_3/L_2 = \sim 4.35$.
The value of Co oxidation state for these L_3/L_2 ratios compared to reference[1] are CoO: 2+, average J14Mn EELS data ~ 2.2+,
In case of Mn L edge,
MnO_2 reference $L_3/L_2 = \sim 1.99$ (This reference data is obtained from the EELS atlas),
average J14Mn EELS data (Figure S3) $L_3/L_2 = \sim 2.382$.
The value of Mn oxidation state for these L_3/L_2 ratios compared to reference[1] are MnO_2: 4+, average J14Mn EELS data ~ 2.9+.

Here are the details of cation vacancy calculations:

For J14Mn, we have 2+ valence for Mg, Ni, Cu, and Zn, while Co and Mn exhibit a mixed 2+ and 3+ valence. We measured the average Co valence to be ~ 2.2+ and Mn to be ~ 2.9+ in J14Mn.

We assume that all cations are in equimolar ratio in J14Mn ($Mg_{1/6}Co_{1/6}Ni_{1/6}Cu_{1/6}Zn_{1/6}Mn_{1/6}O$):

For Mg, Ni, Cu, and Zn with 2+ charge, the cation valence is: $\frac{1}{6} * 2 * 4$
For Co with 2.2+ charge, the cation valence is: $\frac{1}{6} * 2.2$
For Mn with 2.9+ charge, the cation valence is: $\frac{1}{6} * 2.9$

So, this yields the total average cation valence in J14Mn to be:

$$\frac{1}{6} * 2 * 4 + \frac{1}{6} * 2.2 + \frac{1}{6} * 2.9 \ = \ \sim 2.183$$

Since the average charge of cations (A site) is 2.183, which is higher than the oxygen charge, we need to consider vacancies in the cation site (A site) to achieve charge neutrality. Thus, for a rock salt structure $A_{(1-\delta)}O$ with O^{2-}, global charge neutrality requires,

$$(1-\delta)\ x\ 2.183 \ = \ 2$$
$$\delta = 0.0838$$

Thus, we calculate ~8.4% cation vacancies to compensate for the higher average cation valence in J14Mn.

Here are the details of the calculation for J14Cr:
For J14Cr, we have 2+ valence for Mg, Ni, Cu, Co, and Zn, while Cr exhibits 3+ valence.

We assume that all cations are in equimolar ratio in J14Cr ($Mg_{1/6}Co_{1/6}Ni_{1/6}Cu_{1/6}Zn_{1/6}Cr_{1/6}O$):

For Mg, Ni, Cu, Co, and Zn with 2+ charge, the cation valence is: $\frac{1}{6} * 2 * 5$
For Cr with 3+ charge, the cation valence is: $\frac{1}{6} * 3$

So, this yields the total average cation valence in J14Cr to be:

$$\frac{1}{6} * 2 * 5 + \frac{1}{6} * 3 = \ \sim 2.167$$

Since the average charge of cations (A site) is 2.167, which is higher than the oxygen charge, we need to consider vacancies in the cation site (A site) to achieve charge neutrality. Thus, for a rock salt structure $A_{(1-\delta)}O$ with O^{2-}, global charge neutrality requires,

$$(1-\delta)\ x\ 2.167\ =\ 2$$
$$\delta = 0.0771$$

Thus, we calculate ~7.7% cation vacancies to compensate for the higher average cation valence in J14Cr.

References:

(1) Wang, Z. L.; Yin, J. S.; Jiang, Y. D. EELS Analysis of Cation Valence States and Oxygen Vacancies in Magnetic Oxides. *Micron* **2000**, *31* (5), 571–580. https://doi.org/10.1016/S0968-4328(99)00139-0.

(2) Ewels, P.; Sikora, T.; Serin, V.; Ewels, C. P.; Lajaunie, L. A Complete Overhaul of the Electron Energy-Loss Spectroscopy and X-Ray Absorption Spectroscopy Database: Eelsdb.Eu.
